# 80 years of Netherlands Society of Cardiology

**DOI:** 10.1007/s12471-014-0537-9

**Published:** 2014-02-27

**Authors:** V. A. W. M. Umans, E. E. van der Wall

**Affiliations:** 1 Medisch Centrum Alkmaar, Wilhelminalaan 12, 1815 JD Alkmaar, the Netherlands; 2 Interuniversity Cardiology Institute of the Netherlands (ICIN) - Netherlands Heart Institute (NHI), PO Box 19258, 3501 DG Utrecht, the Netherlands

The Netherlands Society of Cardiology (NVVC) was founded on 28 April 1934 at the occasion of the 70th birthday of Professor Karel Frederik Wenckebach. The first record of our Society can be derived from Het Nederlands Tijdschrift voor Geneeskunde (NTvG) 78.III.29, 21 July 1934:3393–3395.

Professor W.A. Kuenen (Leiden) became the Society’s first President and at the same time Wenckebach (Vienna) was appointed Honorary President of the NVVC. In 1934, the NVVC was defined as follows: *The Society is purely scientific, every physician or veterinary surgeon with an interest in cardiology may apply for candidate-membership to the secretary’s office. The annual contribution will amount to five Dutch guilders.*

The first official statutes of the NVVC date back to 1951 when Professor Formyne (Amsterdam) was President. The goals of the NVVC at that time were twofold: 1) *to promote the knowledge of cardiovascular diseases and to take measures for fighting cardiovascular diseases and its consequences, 2) to serve the professional interests of Dutch cardiologists*. In 1949, cardiology was recognised as an independent profession by the Registration Committee for Specialists (currently MSRC). Professor Herman Snellen (Leiden) was the first representative of our profession within the MSRC. Our cardiology training program initially consisted of a 5-year period, including 3 years of internal medicine and 2 years of cardiology. In the 1980s it was decided that our training period would be expanded to a total of 6 years, consisting of 2 years of internal medicine and 4 years of cardiology.

The mission of the NVVC has changed over the years. To fulfil its mission the NVVC has grown into a society that aims to be facilitating, stimulating and guiding. Its policy has become based on three pillars: the patient, public affairs, and the professional. Since 2 years our society is not only focused at cardiologists but also includes allied professionals. The NVVC has intensified relations with partners such as patient organisations (Hart- en Vaatgroep), the Netherlands Heart Foundation, the Society for Cardiovascular Nursing, the Netherlands Society for Cardiothoracic Surgery (NvT), and the European Society of Cardiology (ESC). In this way, the NVVC has become a society for cardiology in general rather that a society for just cardiologists. The time has come to target cardiovascular health instead of cardiovascular disease.

Our 30th anniversary in 1964 was a very memorable year as two prominent members, the Professors Snellen and Van Nieuwenhuizen (Nieuwegein), decided to found the Netherlands Heart Foundation. The Heart Foundation developed into a powerful institute with its main emphasis on prevention, patient information and funding scientific projects. At our 60th anniversary in 1994, the Netherlands Institute for Continuing Cardiovascular Education (CVOI) was established with the main goal to provide education to fellows in training and to cardiologists. The CVOI has proved to be indispensable in educating members of the NVVC and beyond. Certain major achievements have been made during the past 80 years. First of all, since the early 1990s our biannual NVVC congresses have become well-attended meetings creating a true scientific and social platform for all our members. Second, our Website has matured and has become the optimal place to act as a mouthpiece for NVVC members. Next, since 2001 we have our own monthly Society Journal bearing the name the Netherlands Heart Journal (NHJ). The Journal was included in PubMed in 2007 and has been indexed in the Web of Science since 2008. It is expected that the impact factor 2013 of NHJ will pass the threshold of 2.0 [1, 2]. A new initiative by NHJ in 2014 is the installment of an Associate Editors Board recruited from young scientific members from the Interuniversity Cardiology Institute of the Netherlands (young ICIN).

In recent years, our Society has developed from a purely scientific organ to an outstanding professional institute serving a complete spectrum from scientific developments, training and education, generation of guidelines, quality assessment, development of registries, to professional and political interests. Most of these issues are dealt with by our very professional office (Bureau NVVC), taking care of the needs and demands of the members. Many committees and working groups are very active within our Society. Our Society currently contains over 1500 members and the organisational level is close to 100%! As a national society we belong to our parent society, the ESC. In 2013, the NVVC hosted the annual ESC Congress in Amsterdam, which turned out to be a great event both in terms of attendance and scientific quality [3]. At present, our Society is flourishing as never before!

Understandably, we have no recollections of the 10th anniversary of the NVVC (1944) due to the Second World War; neither are there any mementos of the 20th anniversary in 1954. Fortunately, there are still several memorabilia left from the 30th, 40th, 50th, and 60th anniversaries of our Society. All our anniversaries have been celebrated in hotels in Amsterdam: 1974 (Hilton), 1984 (Sonesta Koepelkerk) [4], 1994 (Barbizon Palace), 2004 (Okura Hotel), and 2009 (Mövenpick) [5]. Our 80th anniversary will be celebrated in the RAI Congress Center. At the occasion of our anniversary, a ‘Canon of the Cardiology’ will be presented, which offers a kaleidoscopic view on the achievements in cardiovascular medicine throughout the years with emphasis on the contributions from the Netherlands [6]. Needless to say, our Society can be very proud of past performances from Dutch soil [7, 8]. We wholeheartedly hope you will enjoy our 16th lustrum!
